# Endoscopic and histopathological hints on infections in patients of common variable immunodeficiency disorder with gastrointestinal symptoms

**DOI:** 10.1186/s12876-023-03052-3

**Published:** 2023-11-28

**Authors:** Yang Chen, Yan You, Ji Li, Aiming Yang, Weixun Zhou, Xiaoqing Li

**Affiliations:** 1 Department of Gastroenterology, Peking Union Medical College Hospital, Chinese Academy of Medical Sciences and Peking Union Medical College, Beijing, 100730 China; 2 Department of Pathology, Peking Union Medical College Hospital, Chinese Academy of Medical Sciences and Peking Union Medical College, Beijing, 100730 China

**Keywords:** Common variable immunodeficiency disorder, Endoscopy, Histopathology, Infection, Nodular lymphoid hyperplasia

## Abstract

**Background and aims:**

Common variable immunodeficiency disorder (CVID) patients may have gastrointestinal (GI) involvement and suffer from infections, which are poorly understood. This study aimed to evaluate the clinical, endoscopic, and histopathological features of CVID patients with GI symptoms and determine their correlation with infections.

**Methods:**

We performed a retrospective study on 21 CVID patients with GI symptoms who underwent endoscopic examination in Peking Union Medical College Hospital from 2000 to 2020. The clinical, infectious, endoscopic, and histopathological features were reassessed.

**Results:**

Chronic diarrhea was the most prevalent GI symptom, observed in 95.2% of our CVID cohort. Over 85% of patients had low body weight and malabsorption. Small bowel villous atrophy was found in 90.5% of patients under endoscopy and mostly confirmed by histopathology. GI infections were identified in 9 (42.9%) patients. Of these, 7 patients with diffuse and obvious nodular lymphoid hyperplasia (NLH) of small bowel under endoscopy had significantly higher infection rate (85.7% vs 21.4%, *p* < 0.05), predominantly with *Giardia* and bacteria. Small bowel biopsies showed 95% of patients lacked plasma cells and 60% had increased intraepithelial lymphocytes (IELs), but not significantly different between GI infection and non-infection group. Most patients improved after intravenous immunoglobulin and anti-infection therapy.

**Conclusions:**

CVID could involve GI tract, particularly small bowel. Obvious NLH under endoscopy could be a hint for GI infection in CVID patients. Comprehensive endoscopic and histopathological evaluation may be helpful in CVID diagnosis and identification of potential co-infection, leading to proper treatment.

**Supplementary Information:**

The online version contains supplementary material available at 10.1186/s12876-023-03052-3.

## Introduction

Common variable immunodeficiency (CVID) is the commonest symptomatic primary immunodeficiency with a prevalence ranging from 1/50,000 to 1/200,000. Affected individuals typically manifest with hypogammaglobulinaemia, poor vaccination response, and recurrent infections. The pathogenesis is multifactorial, including several humoral and cell-mediated immunity defects involving B cells, T cells, natural killer cells, macrophages and monocytes, and genetics as well [1, 2]. Given the underlying immunodeficiency heterogeneity, clinical manifestations include recurrent infections, autoimmunity, inflammation, and malignancies. The gastrointestinal (GI) tract which is the largest lymphoid organ is also fairly involved. Common GI symptoms include diarrhea, bloating and abdominal pain. Up to 20% ~ 60% of CVID patients suffer from chronic diarrhea and even malabsorption in severe cases, leading to weight loss, anemia, iron deficiency, loss of minerals and fat-soluble vitamins [1–5]. Importantly, the presence of enteropathy elevates the risk with a 4-fold increase mortality [6]. Why some patients have GI involvement while others do not, remains elusive. Studies on small bowel histopathology in CVID revealed various findings. Most patients, but remarkably not all, lack plasma cells. The major bowel histopathology could be categorized into three patterns: (i) a celiac-like histology, mainly with villous atrophy and increased numbers of intraepithelial lymphocytes (IELs). (ii) IBD-like changes, (iii) nodular lymphoid hyperplasia (NLH) [4, 7, 8]. NLH has been reported in 18% ~ 53% of CVID patients and likely to be underestimated, since asymptomatic subjects may not undergo endoscopy, radiography or biopsy procedures [9–11]. All these conditions might be associated with chronic diarrhea, indicating abnormal immune response in GI tract. There are likely diverse immune-mediated mechanisms resulting in these histological changes, occasionally driven by infections.

CVID patients are usually susceptible to respiratory infections. Meanwhile, recurrent intestinal infections are also common, caused by *Giardia lamblia, Campylobacter* species*, Salmonella* species, cytomegalovirus, norovirus, *Clostridium difficile*, especially in patients with undetectable serum IgA [3, 7, 10, 12, 13]. Intravenous immunoglobulin (IVIG) therapy is insufficient to improve GI symptoms and does not accelerate the response to antibiotics and the eradication of pathogens. IVIG can only substitute IgG, while IgA and IgM, the major secretory antibodies at mucosal surface, cannot be replaced [3, 14, 15]. That made the CVID-associated intestinal infections more challenging.

Endoscopy is helpful for identifying GI mucosal lesions and providing hints on the underlying pathogenic mechanisms. Previous endoscopic studies showed various findings in CVID patients including inflammatory changes (edema, erosion, or ulcer), villous atrophy, NLH, adenoma and even malignancy. NLH may be observed in general population, especially in the ileum of young people, whereas NLH in CVID patients is usually more obvious and widespread, involving the proximal small intestine as well as the distal ileum, even proximal colon. NLH has also been reported to increase the risk of GI tumors (mainly GI lymphoma) and recurrent infections (particularly by *Giardia lamblia*) [16, 17]. So far, there was insufficient evidence to illustrate the correlation among clinical manifestations, infections, endoscopic and histopathological features in CVID patients.

Therefore, we performed a retrospective study in CVID patients with GI symptoms who underwent endoscopy and biopsy, with the aim to reveal the endoscopic and histopathological characteristics as well as their correlation with clinical and immunological features, especially with infections.

## Methods

### Study design and population

#### Ethical aspects

This study was approved by the Institutional Review Board of the Peking Union Medical College Hospital (PUMCH) (No. S-K 2026).

#### Patients

The diagnosis of CVID was according to the European Society for Immunodeficiencies (ESID)/Pan-American Group for Immunodeficiency diagnostic criteria (PAGID) (1999) (patient who has a marked decrease of IgG (at least 2 SD below the mean for age) and a marked decrease in at least one of the isotypes IgM or IgA and fulfills all of the following criteria: onset of immunodeficiency at age > 2 years, absent isohemagglutinins and/or poor response to vaccines, and defined causes of hypogammaglobulinemia have been excluded) [18].

A total of 87 CVID patients were hospitalized from 2000 to 2020 in Peking Union Medical College Hospital. The medical profiles of 21 CVID patients, who had GI symptoms with endoscopy and biopsy histopathology findings, were reviewed retrospectively. Demographic and clinical profiles were collected from inpatient database, including gender, age of onset and CVID diagnosis, GI symptoms (diarrhea, abdominal pain, dyspepsia), body mass index, autoimmune and lymphoproliferative disease, treatment and outcome. Main laboratory data included complete blood count, biochemical tests, serum immunoglobulin levels, lymphocyte subset analysis, D xylose absorption test and stool Sudan III stain for malabsorption. GI infections were identified by stool microscopy for parasites including *Giardia lamblia*, stool toxin for *Clostridium difficile*, as well as stool, mucosal tissue, ascites smear and culture for bacteria. Fungi smears of mucosal leukoplakia were conducted for diagnosing candida esophagitis. CMV DNA and EBV DNA were screened in blood and confirmed by immunohistochemical staining of virus inclusion bodies in colon rectal biopsies*. Helicobacter pylori* (*H. pylori*) infection was confirmed by rapid urease tests.

#### Endoscopic study

A total of 34 endoscopies were performed including 18 upper endoscopies and 16 colonoscopies (13 patients underwent both). The endoscopic images and reports were reviewed from Medicare Endocenter system by two gastroenterologists independently. The endoscopic features of duodenum and terminal ileum, including mucosal edema, villous atrophy and NLH, were recorded by grade. The endoscopic markers suggestive of villous atrophy have been described as loss of circular folds, mosaic pattern, scalloping and nodularity [19]. Mucosa became thinner and submucosal blood vessels were more visible. Local and mild NLH was reported as “+”. Diffuse and obvious NLH was reported as “++”. In addition, 1 capsule endoscopy and 11 GI barium radiographies were performed for small bowel evaluation.

#### Histopathological study

Biopsies were taken from the mucosal lesions and pathological findings were reviewed by two experienced GI pathologists. A total of 50 GI biopsies, including 7 biopsies from stomach, 17 from duodenum, 15 from terminal ileum and 11 from colon, were reevaluated. The presence of plasma cells, villous atrophy, NLH, IELs, acute and/or chronic inflammation (including neutrophil and eosinophilic infiltration), granulomas, crypts distortion, erosion, ulceration, dysplasia, subepithelial collagen deposition, increase in apoptosis and microorganisms were all assessed. Villous atrophy was graded as mild (minor or moderate degrees of shortening and blunting of the villi), marked (short tent-like remainders of the villi) or total (no more villi, flat surface) according to Oberhuber et al. [20]. NLH was defined as reactive lymphoid follicles forming germinal centers in the mucosa and/or submucosa. There was no grading criteria of NLH in histopathology [9, 11]. The percentage of IELs (number of IELs per 100 columnar epithelial cells) was established by counting at least 100 ~ 200 columnar epithelial cells. Small-bowel intraepithelial hyperlymphocytosis was defined by the number of IELs over 30 per 100 columnar epithelial. “Increased” apoptosis was defined as > 1 apoptotic body per 10–15 crypts in small bowel and colon biopsies. Pathogens were detected on Giemsa-stained sections [21].

### Statistical analysis

All statistical analyses were conducted by SPSS 25 (IBM, Armonk, NY). We used mean ± standard deviation for continuous variables with normal distribution, median (*P*_*25*_*, P*_*75*_) for those without normal distribution, and count with percentage for categorical variables. Univariate analyses used parametric (t-tests) or non-parametric methods (Mann–Whitney’s U and Kruskal–Wallis tests) for continuous variables and Fisher’s exact tests for categorical variables. Patients with and without definite GI infections were compared to assess their differences in clinical, immunological, endoscopic, and histopathological characteristics. *P* values are two-sided and considered significant when < 0.05.

## Results

### Demographic, clinical and immunological data

Twenty-one CVID patients with GI symptoms who underwent endoscopy and biopsy are reviewed in Supplementary Table 1. The female-to-male ratio was 0.5. The median age of disease onset was 22 (15, 32) years old and the duration between symptom onset and diagnosis was 2.5 (1, 6) years. Chronic diarrhea was the most common GI symptom, occurring in 95.2% (20/21) of patients, and mainly (80.0%) watery. Moreover, 38.1% of patients suffered from dyspepsia and abdominal pain. 42.9% had fever. 85.0% (17/20, one data missing) had body mass indexes below 18Kg/m^2^. Malabsorption was confirmed in 88.9% (16/18) of patients by D-xylose absorption test. Nutritional anemia was found in 52.4% (11 patients), hypoalbuminemia in 47.6% (10 patients) and 81.0% (17 patients) exhibited hypokalemia and/or hypocalcemia. 28.6% (6 patients) had other autoimmune diseases, such as autoimmune hemolytic anemia, thrombocytopenia, lymphocytic thyroiditis, primary sclerosing cholangitis, vitiligo, and psoriasis.

Details of serum levels of immunoglobins and peripheral blood lymphocytes phenotype at diagnosis are described in Supplementary Table 2. Overall, the median IgG immunoglobulin level was 1.81 (0.37, 2.80) g/L. 95.2% (20 patients) had decreased IgA levels with median 0.07 (0.02, 0.10) g/L. 90.5% (19 patients) had reduced IgM levels with median 0.11 (0.04, 0.16) g/L. CD4 + T cell counts were less than 400/μL in 52.4% (11 patients). An inverted CD4/CD8 ratio was observed in 85.7% (18 patients). 42.9% (9 patients) had B cell counts below 70/*μ*L including two absence of B cell. 81.0% (17 patients) had decreased NK cells counts.

### Infections

42.9% (9 patients) had GI infections with identified pathogens including bacterial infections (4), *Giardia* (3), CMV (2), *Clostridium difficile* (1) and *Candida* (1) (Table 1). Infections in other systems were identified in 71.4% (15 patients). Respiratory and ENT system were most commonly affected. Abdominal CT scans revealed splenomegaly in 52.4% (11 patients) and lymphadenopathy in 47.6% (10 patients). There was no significant difference in clinical manifestations, fever, splenomegaly, lymphadenopathy, and immunological data between GI infection and non-infection patients.

**Table 1:** Endoscopic and histopathological features of small bowel in CVID patients with gastrointestinal symptoms and infections

Case No.	GI infection	Descending duodenum										Terminal ileum									
		Endoscopic features			histopathological features							Endoscopic features			histopathological features						
		mucosal edema	villous atrophy	NLH	villous atrophy	NLH	crypt hyperplasia	IEL (/100 epithelial cells)	plasma cells	Neutrophils(/HPF)	EO(/HPF)	mucosal edema	villous atrophy	NLH	villous atrophy	NLH	crypt hyperplasia	IEL (/100 epithelial cells)	plasma cells	Neutrophils(/HPF)	EO(/HPF)
1		+	–	+	–	+	–	10	–	–	15	ND	ND	ND	ND	ND	ND	ND	ND	ND	ND
2		–	+	+	++	+	–	40	–	+	30	–	+	–	++	+	–	10	–	–	32
3	*Giardia, HP*	–	+	**++**	++	+	–	60	–	–	36	–	+	**++**	++	+	–	35	–	–	21
4	*E.cloacae* (ascites)	++	+	–	ND	ND	ND	ND	ND	ND	ND	–	–	**++**	–	+	–	35	–	+	12
5	*Serratia marcescens, Escherichia coli, Pseudomonas aeruginosa* (ileum tissue culture)	++	+	**++**	+++	+	ND	> 30	–	ND	ND	++	+	+	+++	+	ND	> 30	–	ND	ND
6		ND	ND	ND	ND	ND	ND	ND	ND	ND	ND	–	+	+	–	+	–	5	–	–	5
7		+	+	–	++	–	+	70	–	–	15	ND	ND	ND	ND	ND	ND	ND	ND	ND	ND
8		+	–	–	–	–	–	10	–	–	40	–	–	–	–	–	–	27	–	–	15
9	*Clostridium difficile*	+	+	**++**	++	+	+	50	–	–	8	+	+	**++**	+	+	–	35	–	+	18
10		+	+	–	++	+	+	30	–	+	50	+	+	–	++	+	–	45	–	+	48
11		+	+	**++**	ND	+	ND	ND	ND	ND	ND	ND	ND	ND	ND	ND	ND	ND	ND	ND	ND
12		ND	ND	ND	ND	ND	ND	ND	ND	ND	ND	+	+	–	+	+	–	20	increased	+	15
13	*Vibrio*	+	+	–	++	–	+	25	–	+	7	–	+	–	++	–	+	15	–	+	10
14		–	+	–	++	+	–	35	–	–	10	–	+	–	++	+	–	35	–	–	17
15	candida esophagitis	+	+	+	++	–	+	100	–	+	18	–	+	+	++	+	+	60	–	+	10
16		++	+	–	+	–	+	60	–	+	10	ND	ND	ND	ND	ND	ND	ND	ND	ND	ND
17	CMV	+, ulcers	+	–	++	–	+	25	–	+	15	ND	ND	ND	ND	ND	ND	ND	ND	ND	ND
18		++	+	–	++	+	+	55	–	–	20	+	+	+	+	+	–	32	–	–	11
19	CMV, *Giardia, HP*	ND	ND	ND	ND	ND	ND	ND	ND	ND	ND	+, ulcers	+	**++**	–	+	–	20	–	+	10
20		+	+	–	++	–	+	50	–	+	5	ND	ND	ND	ND	ND	ND	ND	ND	ND	ND
21	*Giardia*	+	+	**++**	+++	+	+	25	–	+	10	–	+	**++**	+++	+	–	20	–	+	15

-, abscent, +, present, *ND* not done

*EO* eosinophils in lamina propria

*NLH* nodular lymphoid hyperplasia: under endoscopy, + focal and mild, ++ diffuse and obvious

histopathological villous atrophy: + blunt, ++partial, +++total

### Endoscopic and histological findings

#### Small bowel

**Fig. 1:**
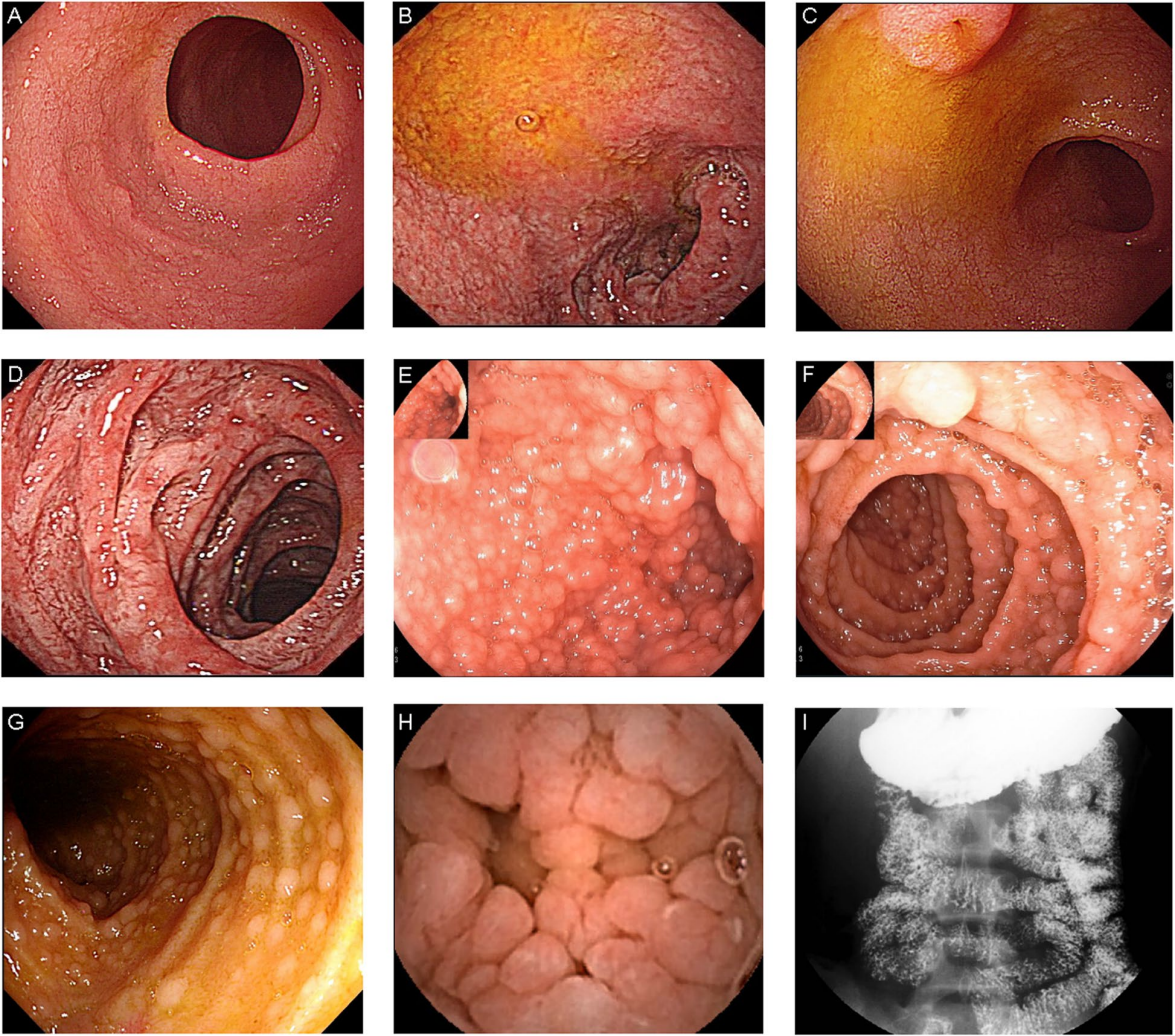
The endoscopic and GI barium enterographic features of small bowel in CVID patients with GI symptoms. **A**, **B** mucosal edema and villous atrophy of duodenal bulb. **C**, **D** Mucosal edema and villous atrophy of descending duodenum. **E, F, G** NLH of duodenal bulb, descending part, and terminal ileum. A pseudo-polypoid pattern of mucosa was observed. **H** NLH of jejunum under capsule endoscopy. **I** Oral enterography showed diffuse nodular filling defects which were confirmed as NLH

**Fig. 2:**
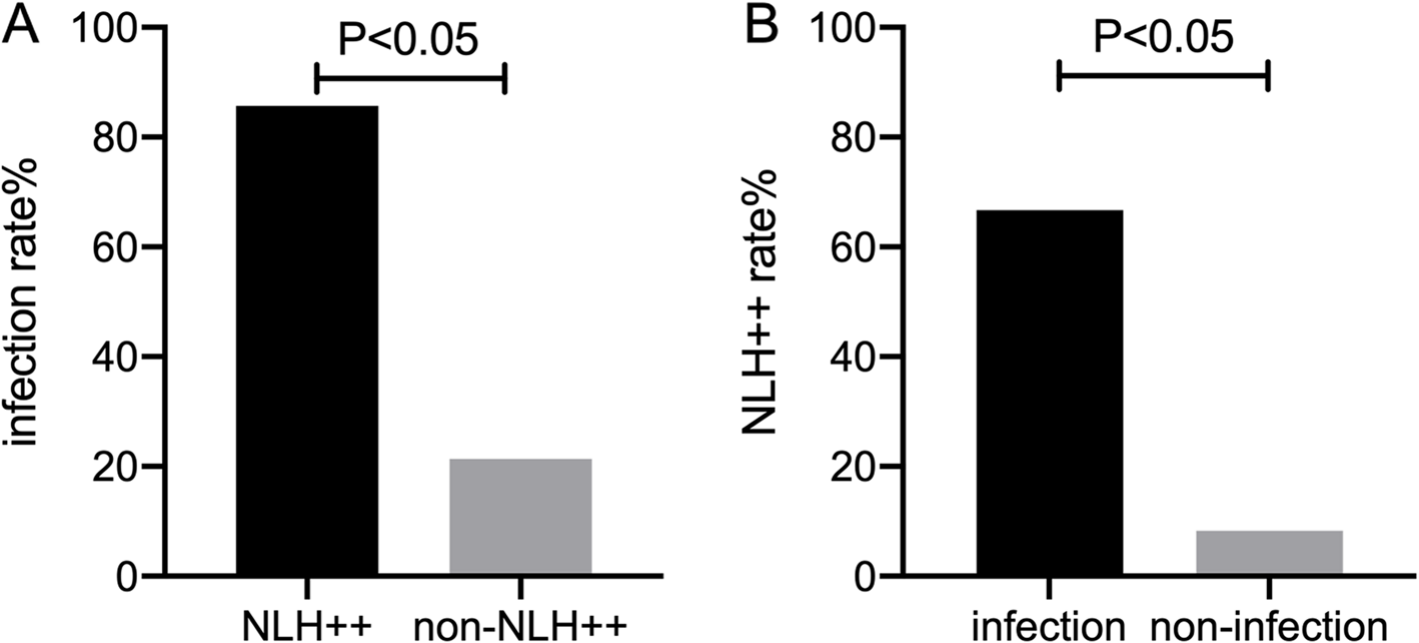
The probable correlation between diffuse and obvious NLH under endoscopy and infections. **A** The patients with diffuse and obvious NLH (shown as NLH++) under endoscopy had higher infection rate. **B** The patients with GI infections had higher rate of obvious and diffuse NLH (shown as NLH++)

The endoscopic and histopathological features are shown in Table 1 and Fig. 1. The duodenal mucosal edema and villous atrophy were found in 83.3% (15/18) and 88.9% (16/18) patients by upper endoscopy respectively. Mucosal edema and villous atrophy of terminal ileum were found in 40.0% (6/15) and 86.7% (13/15) patients by colonoscopy respectively. 90.5% (19/21) patients had small bowel villous atrophy under endoscopy. NLH was detected in 44.4% (8/18) patients’ duodenum (including 5 diffuse and obvious NLH) and 60.0% (9/15) patients’ terminal ileum (including 5 diffuse and obvious NLH). In all, 57.1% (12/21) patients had small bowel NLH and 7 of them were diffuse and obvious recorded as“++”. The patients with NLH++ had significantly higher infection rate than others (85.7% vs 21.4%, *p* < 0.05), including three *Giardia* infections (Fig. 2A). However, the patient only with CMV infection did not have obvious NLH, but ulcer. From another point of view, the patients with definite GI infection had higher prevalence of NLH++ compared with non-infection patients (66.7% vs 8.3%, *p* < 0.05) (Fig. 2B). The barium radiography showed nodular filling defect in 27.3% (3/11) patients and rough mucosa in 36.4% (4/11) patients. Capsule endoscopy of one patient with *Giardia* infection had diffuse NLH all through the small bowel.

**Fig. 3:**
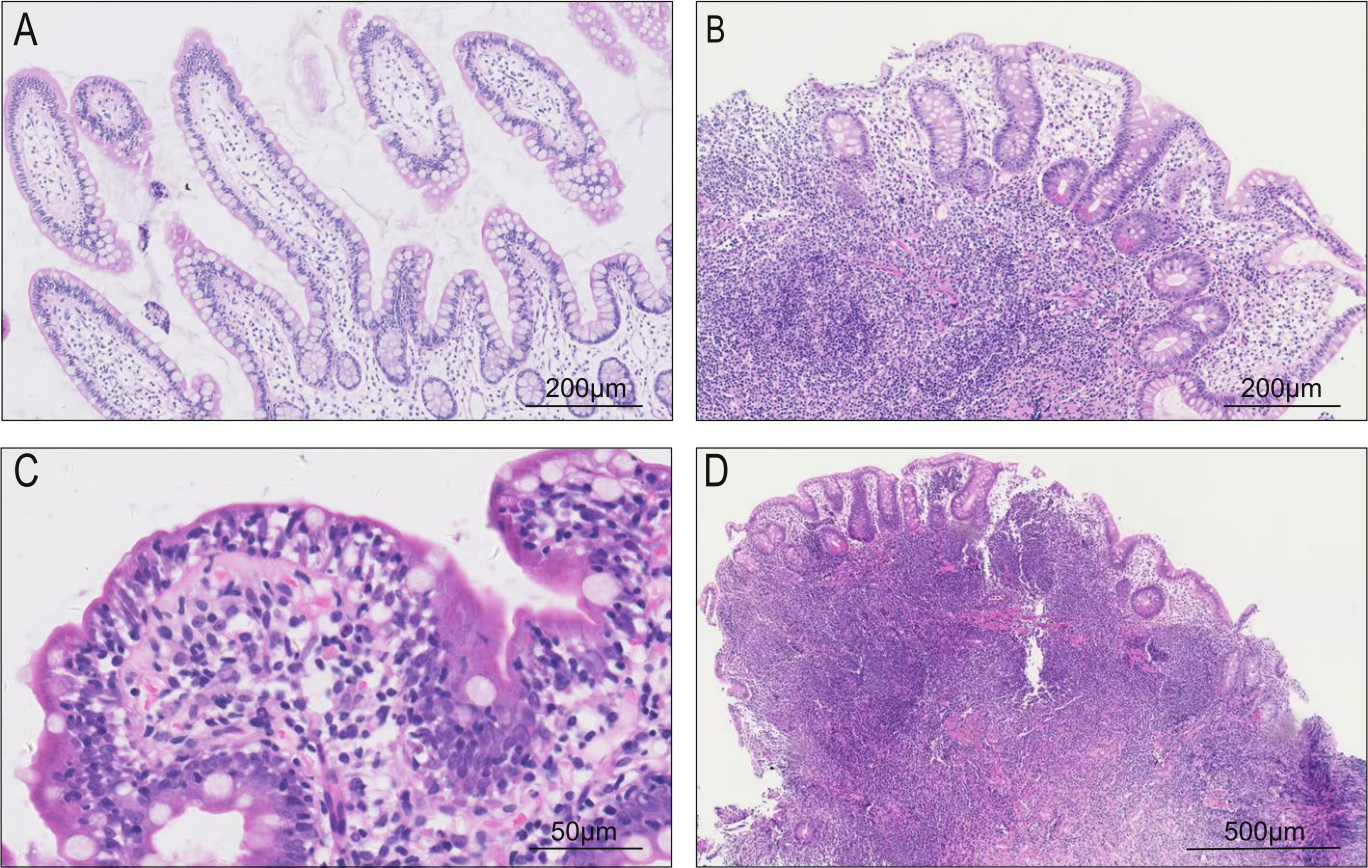
The histopathological features of small bowel in CVID patients with gastrointestinal symptoms. **A** Normal villi of small bowel in healthy people (H&E; original magnification, ×100). **B** Villous atrophy of small bowel in CVID patients (H&E; original magnification, × 100). **C** Increased IELs and plasma cell deficiency of small bowel in CVID patients (H&E; original magnification, ×400). **D** NLH localized at mucosa and submucosa of small bowel in CVID patients (H&E; original magnification, × 50)

Duodenal biopsies from 17 patients and terminal ileum biopsies from 15 patients were reassessed (Fig. 3). The histopathology confirmed duodenal villous atrophy in 87.5% (14/16) patients and ileum mucosal atrophy in 73.3% (11/15). Only two biopsies did not match endoscopic findings (atrophy under endoscopy but not in histopathology of ileum, might be due to the biopsy position). NLH was detected in duodenum of 58.8% (10/17) patients and in terminal ileum of 86.7% (13/15) patients. Seven biopsies with NLH did not show gross appearance under endoscopy. 95% (19/20) patients lacked plasma cells. 60.0% (12/20) patients had increased IELs. There were no significant differences in the incidences of NLH and increased IELs by histopathology between GI infection and non-infection patients.

#### Stomach

Only one was diagnosed as atrophic gastritis and others without obvious gastric atrophy. The most common sign was mucosa edema observed in 8 patients and multiple ulcers were seen in one patient with CMV infection. Two patients had *H. pylori* infections including one with antral nodularity.

Six biopsies were obtained from gastric antrum and one from gastric body. Histopathology showed acute and/or chronic gastritis in 5 patients whose plasma cells were also absent. NLH could be seen in one patient who had nodular gastritis with both *H. pylori* and *Giardia* infections. IELs did not increase in all biopsy samples.

#### Colon

Sixteen colonoscopies were performed with cecal intubation rate of 93.8% except one finally intubated to hepatic flexure. Three patients had colon mucosal erosion and/or ulcer including one CMV infection and one vibro infection. Two patients had colon mucosal edema. Biopsies were performed on patients above and 3 patients with normal appearance of colon under endoscopy.

Eleven biopsies were obtained from 8 patients. All patients had lymphocytes infiltrated in lamina propria in different degree without increased IELs. Four patients had neutrophils infiltrated in epithelial or lamina propria. Plasma cells were absent in 5 patients. 75.0% of patients had focal crypt distortion.

### Treatment and outcome

85.7% (18 patients) were treated with antibiotics, in which 9 patients had identified pathogens and 2 patients received ganciclovir for CMV infection. IVIG therapy was given to 14 patients. Clinical symptoms improved in 90.5% (19 patients).

## Discussion

We retrospectively reviewed 21 CVID patients with GI symptoms who underwent endoscopy and biopsy. The key findings included: (i) Chronic diarrhea with malabsorption was the predominant clinical manifestation of CVID enteropathy. (ii) Small bowel was mainly affected with distinctive endoscopic features such as mucosal edema, villous atrophy, and extensive NLH. Accordingly, the histopathology revealed villous atrophy, increased IELs, NLH, and decreased plasma cells in duodenum and terminal ileum mucosa. (iii) Diffuse and obvious NLH may be an endoscopic sign of infections, especially for *Giardia* and bacteria.

In our CVID cohort, 23% (20/87) of patients experienced diarrhea, with a high malabsorption rate as determined by D-xylose absorption test. Quinti I reported chronic diarrhea was observed in 22.4% of CVID patients and resulted in a significant malabsorption in 8.1% [5]. Chapel H reported 9% CVID enteropathy, which was defined as biopsy-proven lymphocytic infiltration in lamina propria and interepithelial mucous with villous atrophy, insensitive to gluten withdrawal [6]. However, CVID enteropathy lacks universal definition, with estimated prevalence of 9% ~ 34% [22]. Endoscopy could give a general clue for villous atrophy with confirmation by histopathology [7, 19]. Previous studies found intestinal villous atrophy in approximately 30% ~ 50% of CVID patients with GI symptoms [21–23]. We found he duodenal villous atrophy was over 85%, which was higher than previous reported. IM Andersen categorized CVID enteropathy into severe and non-severe types based on whether there were weight loss, malnutrition, and severe GI-loss [22]. In our cohort, 85% of patients were under weight, and 52% presented with nutritional anemia, suggesting severe disease manifestation. Some disparities in findings might arise due to sample size limitations, disease severity, and regional factors. Other conditions displaying villous atrophy need to be differentiated including celiac disease, autoimmune enteropathy, tropical sprue, protracted viral or bacterial infection, giardiasis, T-cell lymphoma, food protein hypersensitivity, and graft-versus-host disease [20, 24]. CVID patients have hypoimmunoglobins, profoundly decreased plasma cells and NLH in mucosa, and resistance to gluten-free diet [21, 23]. So, the clinical history, biopsy histopathology and therapy response could help to get accurate diagnosis.

Further differentiation is required when CVID enteropathy presents with hypoalbuminemia from conditions like protein loss enteropathy (PLE). PLE can be caused by different mechanisms: increased lymphatic pressure, mucosal erosions, and increased mucosal permeability. It has been reported that PLE has lower infection and lymphoproliferation rate, higher serum levels of IgG, and mildly decreased to normal serum levels of IgA (> 0.5 g/L) than CVID. However, PLE can occur during CVID and requires higher IgG replacement therapy dosage [25]. There are 10 patients with hypoalbuminemia in our study, who all had obvious low IgA levels. Nine of them lacked plasma cells in gut biopsy, and the other one with mild hypoalbuminemia had no etiology of PLE. All above support their CVID diagnosis.

NLH shows pseudopolypoid appearance with multiple or occasionally innumerable nodules measuring 2–3 mm and usually not exceeding 10 mm in diameter of duodenum or ileum mucosa under endoscopy, sometimes through the whole small bowel [4, 10, 11, 23, 26]. Biopsy could confirm the diagnosis of NLH in microscopic view, however, it is dependent on the biopsy site and hard to evaluate the gross degree. Endoscopy is a good compensatory tool for the general evaluation. NLH had been reported in following conditions: CVID, selective IgA deficiency syndrome, giardiasis, *H. pylori* infection (gastric-NLH), food hypersensitivity, HIV, familial adenomatous polyposis, and GI malignancy, especially lymphoma [9, 17, 26]. Our data suggests that diffuse and obvious NLH may indicate infections, especially with *Giardia* and bacteria. A previous study on infections in 252 CVID patients showed that 47% had GI symptoms, 14% had *Giardia lamblia* infection and 19% had other GI bacterial infections [13]. *Giardia lamblia* could be one of the antigenic stimulators and associated with NLH in patients with or without immunodeficiency syndromes, leading to watery diarrhea, steatorrhea, and malabsorption [17, 27–29]. It is reported that diffuse NLH of the bowel associated with CVID and refractory giardiasis markedly improved after successfully treating giardiasis [30]. The pathogenesis of NLH is still unknown. Infection maybe a trigger of mucosal immune response and disturbance. Repetitive stimulation of infectious agents probably lead to the hyperplasia of lymphoid follicles [9]. Lymphoproliferation was also present elsewhere in CVID, such as splenomegaly and lymphadenopathy. Similarly, *H. pylori* infection could cause gastric nodularity which could be normal after *H. pylori* eradication treatment. Increased IELs might also be an immune compensation and dysregulation. In all, infections could cause both acute diarrhea and chronic immune dysregulation in CVID enteropathy. Our study showed that diffuse and obvious NLH might be an endoscopic clue for infections in these patients. Although NLH may be related to high risk of malignancy, we do not find in our study.

The reason for the variance in CVID manifestations, with some patients developing GI symptoms and others not, remains unclear. T-cell dysfunction and autoimmunity against intestinal tissue, absence of mucosal plasma cells and defective antibody production, especially mucosal IgA, have been reported [3]. On the other hand, infections maybe also an important factor such as chronic norovirus [22]. Early diagnosis and IVIG replacement therapy (0.4 to 0.5 g/kg/month) can reduce the incidence of respiratory tract infection. However, IVIG did not improve diarrhea, especially in patients with lower serum IgA titers. Different options have been used to treat GI symptoms, for example, antibiotics such as metronidazole or ciprofloxacin, 5-aminosalicylic acid, and immunosuppressive agents such as corticosteroids, azathioprine, and infliximab [2, 12]. Endoscopy is a good general evaluation providing useful information for mucosal change and possible co-infections. The endoscopic and histopathological assessment should be performed in CVID with GI symptoms to facilitate diagnosis and treatment.

## Limitations

Our study has several limitations. Firstly, it was a single center retrospective study with potential biases due to a limited number of patients. Secondly, we utilized the ESID/PAGID (1999) criteria for CVID, while more recent updates have been made. However, these were all phenotypic diagnostic criteria, and there will be no definite CVID diagnoses, until the genetic or other etiopathogenetic causes can be defined for all patients [31, 32]. It was reported that the probable disease-causing mutations were found in 30% CVID patients by whole exome sequencing (WES) [33]. The gene analyses (WES, whole genome sequencing, or panel) was not performed routinely in our clinic. We did not know whether the patients had monogenic diseases, which might be done in the future. Thirdly, we lacked consistent laboratory data across time, but ensured expert reassessment of endoscopic and histopathological findings. Finally, the improvement was evaluated by clinical symptoms. We could not evaluate endoscopic and histopathological features from patients without GI symptoms or those post-treatment.

## Conclusion

CVID frequently affects the GI tract, the biggest immune and environmental interface. The precise role of infections remains uncertain but is believed to potentially trigger immune regulation and impact on disease progression. Endoscopic evaluation, especially on small intestine, is crucial for CVID patients with GI symptoms. The diffuse and obvious NLH may be a sign for GI infection. Comprehensive endoscopy and histopathology assessment can offer vital diagnostic clues for CVID enteropathy and infections, facilitate prompt treatment, and ultimately improve patient quality of life. Further research is needed to refine treatments for this patient group.

### Supplementary Information

**Additional file 1.**

**Additional file 2.**

## Data Availability

The datasets used and/or analyzed during the current study available from the corresponding author on reasonable request.

## Abbreviations

CVID: Common variable immunodeficiency disorder
GI: Gastrointestinal
*H. pylori*: *Helicobacter pylori*
IELs: Increased intraepithelial lymphocytes
IVIG: Intravenous immunoglobulin
NLH: Nodular lymphoid hyperplasia
WES: Whole exome sequencing
