# Correction: Endoscopic and histopathological hints on infections in patients of common variable immunodeficiency disorder with gastrointestinal symptoms

**DOI:** 10.1186/s12876-023-03098-3

**Published:** 2024-01-04

**Authors:** Yang Chen, Yan You, Ji Li, Aiming Yang, Weixun Zhou, Xiaoqing Li

**Affiliations:** 1 Department of Gastroenterology, Peking Union Medical College Hospital, Chinese Academy of Medical Sciences and Peking Union Medical College, Beijing, 100730 China; 2 Department of Pathology, Peking Union Medical College Hospital, Chinese Academy of Medical Sciences and Peking Union Medical College, Beijing, 100730 China

**Correction: BMC Gastroenterol 23, 413 (2023)**

10.1186/s12876-023-03052-3

Following publication of the original article [1], the authors reported several typographical errors in the article body. The corrections are as follows:

1. In the header of Table 1 ‘ilum’ has been corrected to ‘ileum’.
2. In a sub-header of columns 3 and 13 of Table 1 ‘mucous edema’ has been corrected to ‘mucosal edema’.
3. In the third paragraph of the ‘Discussion’ section ‘Nine of them lack plasma cells in gut biopsy, and the other one with mild hypoglycaemia had no etiology of PLE’ has been corrected to ‘Nine of them lacked plasma cells in gut biopsy, and the other one with mild hypoalbuminemia had no etiology of PLE’.
4. In the fourth paragraph of the ‘Discussion’ section ‘NLH shows pseudopolypoid appearance with multiple or occasionally innumerable nodules measuring 2–3 mm and usually not exceeding 10 mm in diameter of duodenum or ileum mucous under endoscopy…’ has been corrected to ‘NLH shows pseudopolypoid appearance with multiple or occasionally innumerable nodules measuring 2–3 mm and usually not exceeding 10 mm in diameter of duodenum or ileum mucosa under endoscopy…’.

The original article has been updated.
